# Obesity, Preserved Ejection Fraction Heart Failure, and Left Ventricular Remodeling

**DOI:** 10.3390/jcm12093341

**Published:** 2023-05-08

**Authors:** Jason Stencel, Hamid R. Alai, Aneesh Dhore-patil, Daniela Urina-Jassir, Thierry H. Le Jemtel

**Affiliations:** 1 Section of Cardiology, John W. Deming Department of Medicine, Tulane University School of Medicine, Tulane University Heart and Vascular Institute, New Orleans, LA 70112, USA; jstencel@tulane.edu (J.S.); halai@tulane.edu (H.R.A.); adhore@tulane.edu (A.D.-p.); durinajassir@tulane.edu (D.U.-J.); 2 Southeast Louisiana VA Healthcare System (SLVHCS), New Orleans, LA 70119, USA

**Keywords:** obesity, HFpEF, metabolic bariatric surgery, left ventricular mass, epicardial adipose tissue

## Abstract

Owing to the overwhelming obesity epidemic, preserved ejection fraction heart failure commonly ensues in patients with severe obesity and the obese phenotype of preserved ejection fraction heart failure is now commonplace in clinical practice. Severe obesity and preserved ejection fraction heart failure share congruent cardiovascular, immune, and renal derangements that make it difficult to ascertain whether the obese phenotype of preserved ejection fraction heart failure is the convergence of two highly prevalent conditions or severe obesity enables the development and progression of the syndrome of preserved ejection fraction heart failure. Nevertheless, the obese phenotype of preserved ejection fraction heart failure provides a unique opportunity to assess whether sustained and sizeable loss of excess body weight via metabolic bariatric surgery reverses the concentric left ventricular remodeling that patients with preserved ejection fraction heart failure commonly display.

## 1. Introduction

Over the past 3 decades, body-mass index (BMI, kg/m^2^) has increased uncontrollably in the United States (US). By 2030, half of US adults will have a BMI ≥ 30 and a quarter will have a BMI ≥ 40 [1,2,3]. Recent trials and investigations of preserved ejection fraction heart failure (HFpEF) embody the high prevalence of obesity. Mean BMI ranges from 27.6 to 42.4 in 17 randomized controlled trials of HFpEF and is ≥30.0 in 12 of the 17 trials [4,5,6,7,8,9,10,11,12,13,14,15,16,17,18,19,20,21]. Mean BMI is 32.4 in 11 recent studies of HFpEF and is 38.6 in the 6 studies concerning obesity and HFpEF [19,22,23,24,25,26,27,28,29,30,31,32,33,34,35]. Hence, HFpEF commonly ensues in patients with BMI ≥ 30 and the obese phenotype of HFpEF syndrome is increasingly prevalent in clinical practice [36,37]. In addition to tight control of blood pressure (BP) and volume overload, loss of body weight through metabolic bariatric surgery (MBS), or novel antiobesity medications may improve outcomes in patients with the obese phenotype of HFpEF. 

We highlight how congruent are the derangements of HFpEF and obesity on the cardiovascular system, functional capacity, immune system, and renal function. The effects of obesity on cardiac metabolism and obesity-related conditions (hypertension [HT] and type 2 diabetes [T2D]) on HFpEF were recently reviewed and thus not discussed [38,39,40,41,42]. We then examine the effect of MBS on left ventricular mass (LVM) assessed by cardiac magnetic resonance imaging (CMR) and the effect of MBS on the incidence of heart failure (HF) in patients with obesity. Last, we outline the subset of patients with HFpEF and obesity who may experience the most reduction in LVM and improvement in LV diastolic dysfunction (LVDD) after MBS or effective antiobesity pharmacologic treatment. 

The extent of LV remodeling reversal, as evidenced by LVM reduction and LVDD improvement after a large loss of body weight, will help assess the contribution of excess body weight to the obese phenotype of HFpEF. 

## 2. Cardiac Remodeling and Functional Capacity

### 2.1. Preserved Ejection Fraction Heart Failure

Concentric left ventricular (LV) remodeling, right ventricular (RV) dilatation/dysfunction, and epicardial adipose tissue (EAT) thickness are more prominent in HFpEF patients with a BMI ≥ 35 than in patients with a BMI ≤ 30 [29]. With larger ventricular volumes and increased EAT thickness, HFpEF patients with BMI ≥ 35 display greater cardiac restraint and ventricular interdependence than their counterparts with BMI ≤ 30 [29,43]. Exercise-induced pulmonary hypertension (PHT) is greater and pulmonary artery vasodilator reserve is lower in HFpEF patients with BMI ≥ 35 than ≤30 [29]. When evaluated by peak oxygen uptake (V_O2_, ml/min/kg), functional capacity is lower in HFpEF patients with BMI ≥ 35 than ≤30 [29]. Peak V_O2_ correlates more closely with LV filling pressure than with LV diastolic dysfunction in HFpEF patients with an average BMI of 38.4 [44]. The thickness of EAT is inversely related to peak V_O2_ after adjustment for pulmonary vascular resistance in HFpEF patients [45,46]. Whether skeletal muscle O_2_ transport and utilization impair functional capacity remains unsettled in HFpEF [19,47,48,49]. Peak V_O2_ correlates inversely with the amount of central and intermuscular adipose tissue (AT) in elderly HFpEF patients [31]. Visceral adipose tissue (VAT), a marker of central adiposity, was associated with concentric LV remodeling in the Dallas Heart Study and the Multiethnic Study of Atherosclerosis (MESA) [50,51]. Further, VAT was a strong risk factor for incident HFpEF in the SCReening Evaluation of the Evolution of New Heart Failure (SCREEN-HF) study and the Multiethnic Study of Atherosclerosis (MESA) [52,53]. Last, the VAT area closely correlates with the degree of hemodynamic impairment in women with HFpEF [54]. 

### 2.2. Obesity

Thirty-two years ago, the Framingham Heart Study uncovered the association between obesity and LVM after adjustment for age and BP [55]. Over the next 3 decades, numerous observational and community-based studies corroborated the association between obesity and LV remodeling [56,57,58,59,60,61,62,63,64,65,66,67,68]. Concentric remodeling (normal LVM/volume ratio) and concentric hypertrophy (increased LVM/volume ratio are common patterns of LV remodeling in patients with BMI ≥ 30 [64,69]. Body-fat distribution affects patterns of LV remodeling. In the Dallas Heart Study, participants with BMI ≥ 30 displayed LV remodeling with central adiposity linked to concentric hypertrophy and lower-body adiposity to eccentric hypertrophy [50,70]. Adverse LV remodeling is a major mediator of obesity-outcome associations in the UK Biobank participants [71]. Expanding EAT may contribute to LV remodeling by increasing septal thickness [72]. However, whether EAT independently promotes LV remodeling and affects outcomes in HFpEF awaits further studies [73,74]. 

Middle-aged and older patients with BMI ≥ 30 have an increased incidence of RV hypertrophy in the absence of traditional risk factors like obstructive sleep apnea (OSA), PHT, and obstructive lung disease [75,76]. Obesity-related RV remodeling is sex-specific. Women have mixed concentric and eccentric RV remodeling with high ejection fraction (EF) while men have concentric remodeling with larger RV volumes than women [77]. The underpinnings of the association between obesity and RV remodeling are incompletely understood. In addition to increased RV afterload due to obesity-related LVDD, circulating cytokines, growth hormones, adipokines (leptin, Ang II, insulin, and aldosterone), BP, and nocturnal hypoxemia contribute to obesity-related RV remodeling [59,77,78]. 

High systolic BP and arterial stiffening lead to ventricular-arterial uncoupling, afterload mismatch, and LV remodeling/hypertrophy [79,80]. Increased EAT thickness contributes to arterial stiffening in HFpEF [81]. Hypertension and age are the leading conditions that promote LV remodeling/hypertrophy [82]. However, aggressive antihypertensive therapy did not preclude the development of LV hypertrophy in the Campania Salute Network study whereas loss of body weight led to a reduction of LV mass [83,84,85]. Modest weight loss was shown to reduce LVM independent of BP changes in overweight patients with HT, and the Bogalusa Heart Study unveiled a stronger association between LVM and BMI than BP and BMI [65,86]. 

**Figure 1:**
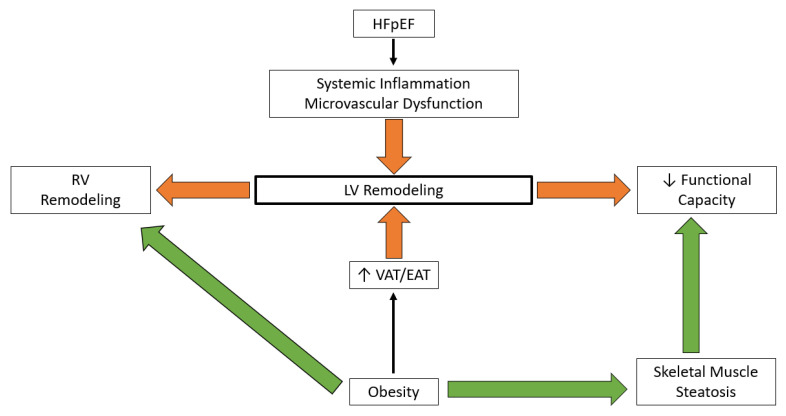
Cardiac remodeling and functional capacity: HFpEF—Heart failure with preserved ejection fraction, LV—Left ventricle, RV—Right ventricle, VAT—Visceral adipose tissue, EAT—Epicardial adipose tissue.

In patients without disability or overt pulmonary obstructive disease, functional capacity estimated by the 6 min walk test, distance decreases as BMI increases (Table 1) [87,88]. The leveling of the 6 min walking distance when BMI is ≥35 suggests a tipping point in obesity where functional capacity is severely limited and further weight gain has only an incremental effect on mobility [89] (Figure 1).

## 3. Inflammation

### 3.1. Preserved Ejection Fraction Heart Failure

Inflammation plays a major role in the development and progression of HFpEF [90]. Circulating levels of inflammatory proteins correlated with the degree of LV stiffness when controlled for the burden of comorbidities in the prevalence and correlates of coronary microvascular dysfunction in an HFpEF (PROMIS-HFpEF) study [91]. Inflammatory biomarkers like interleukin (IL)-6, tumor necrosis factor (TNF)-a, and C-reactive protein (CRP) independently predict the incidence of HFpEF after adjustment for clinical and laboratory covariates [92]. Deletion of IL-6 attenuates transverse aortic constriction-induced LV remodeling and hypertrophy in mice [93]. Patients with HFpEF have increased concentrations of circulating inflammatory monocytes and healthy monocytes acquire profibrotic/M2 macrophage features when exposed to the serum of HFpEF patients [94]. A heightened level of systemic inflammation may promote myocardial infiltration of C-C chemokine receptor 2 (CCR2+) monocytes that release inflammatory cytokines leading to fibroblast activation and subsequent fibrosis [95]. Myocardial monocyte infiltration is highly prevalent in HFpEF and correlates with age and renal disease [96]. Cardiac inflammatory cells contribute to LVDD through the release of transforming growth factor (TGF)-b and remodeling of the extracellular matrix in patients with normal EF [97]. Although inflammation aggravates metabolic stress in the high-fat diet+ L-NAME model, immune modulation therapy is so far underwhelming in patients with HFpEF [98]. Whether obesity amplifies the myocardial, inflammatory, and metabolic derangements in HFpEF or fosters new derangements is being investigated in cardiometabolic models of HFpEF like the Goto-Kakizaki, obese ZSF1 rat, the high-fat diet + L-NAME mice and the WD + DOCA swine [98,99]. 

### 3.2. Obesity

Low-grade systemic inflammatory processes and related oxidative stress are the hallmarks of obesity [100]. When expanding, white adipose tissue (WAT) switches from an anti-inflammatory to a proinflammatory response [101,102]. The proinflammatory response is stronger in visceral depots (VAT, EAT), where only hypertrophy mediates expansion than in subcutaneous adipose tissue (SAT) where both hypertrophy and hyperplasia mediate expansion [103]. WAT contains more macrophages in visceral than peripheral depots [104]. Ectopic lipid deposition in the liver and skeletal muscle dampens peripheral insulin signaling and results in VAT inflammation, along with insulin resistance and subsequent T2D [105]. Fatty acids can promote inflammation directly via toll-like receptor (TLR) TLR4 and TLR2 through protein fetuin A resulting in nuclear factor-k B (NFkB) and c-Jun kinase (JNK) activation [106]. Uncoupled cellular respiration in obesity leads to adipose-cell hypoxia that initiates an inflammatory state via the hypoxia-inducible factor 1a gene program [107]. Adipocyte necrosis activates an intracellular multiprotein signaling complex with nucleotide-binding leucine-rich repeat-containing receptor 3 (NLRP3 inflammasome) that leads to the activation of caspase1 and secretion of proinflammatory IL-1b [108]. Adipocyte necrosis, a byproduct of increased cell turnover in obesity, is closely associated with chronic inflammation in the development of crown-like structures (CLS) [109]. Last, mechanical stresses, interaction with the extracellular matrix, and mitochondrial dysfunction may trigger adipocyte inflammation [110]. 

Visceral adiposity promotes local and systemic inflammation, oxidative stress, abnormal lipid metabolism, vascular endothelium dysfunction, thrombosis, and insulin resistance that hastens the development of atherosclerotic cardiovascular disease [111]. Adaptative immunity, particularly T-lymphocytes, promotes microvascular remodeling [112]. Obesity, particularly VAT expansion, increases cardiovascular risk [113,114]. However, quantification of VAT area or volume requires computerized tomography (CT) or magnetic resonance imaging (MRI) and thus is not routinely used in clinical practice for prognostication. Whether increased EAT thickness by echocardiography is a convenient surrogate marker of VAT expansion or an independent driver of HFpEF remains to be determined [72,115,116]. 

## 4. Circulation

### 4.1. Preserved Ejection Fraction Heart Failure

#### 4.1.1. Coronary Artery Disease

Though impaired LV relaxation is an early and long-lasting sign of myocardial ischemia, diagnosis, and treatment of CAD, it is not as steadfastly pursued in HFpEF as in HFrEF [117]. The mean prevalence of CAD in HFpEF ranges from 20–76%, dependent on clinical settings, with a median prevalence of 41% [118]. A history of myocardial infarction (MI) and extensive CAD increases the incidence of HFpEF independent of recurrent MI [119,120,121]. After adjustment for common cardiac comorbidities, CAD was a risk factor for incident HFpEF in the Atherosclerosis Risk in Communities (ARIC) study [122]. Patients with CAD and HFpEF were at higher risk of all-cause mortality and sudden cardiac death than their counterparts without CAD in the Irbesartan in HFpEF (I-PRESERVE) study [123]. Coronary microvascular dysfunction (CMD), whether endothelium independent or dependent, was more prevalent than epicardial CAD in a multicenter cohort of 106 patients hospitalized for HFpEF [124]. In the absence of epicardial CAD, up to 80% of HFpEF patients have CMD [124].

#### 4.1.2. Microvasculature

HFpEF patients have significant microvasculature dysfunction, even in the absence of obstructive epicardial atherosclerotic disease. Patients with HFpEF and no obstructive CAD have a 2.62-fold lower myocardial flow reserve (MFR, calculated as stress versus rest by Rb-82 cardiac positron tomography imaging) than hypertensive and nonhypertensive controls [125]. Invasive assessment of coronary flow reserve (CFR) and index of microvascular resistance (IMR) during cardiac catheterization have shown that 36.7% of HFpEF patients have both abnormal CFR and IMR and another 36.7% have either abnormal CFR or IMR [126]. Microvascular complications are more prevalent in patients with HFpEF and T2D and are associated with greater LV hypertrophy/remodeling and worse quality of life than in patients with HFpEF and no T2D [127]. Coronary and systemic microvascular dysfunction increases the risk of all-cause mortality and HF hospitalization in patients with HFpEF [128]. 

#### 4.1.3. Coronary Vascular Endothelium

The contribution of endothelium-independent and endothelium-dependent vasodilatation to CMD ranges from balanced to mostly endothelium-independent in HFpEF [124,128,129,130]. Further, in addition to functional microvascular derangements, coronary microvascular rarefaction reduces myocardial O_2_ delivery in HFpEF [131]. Stress CMR shows that abnormal subendocardial perfusion correlates with LVDD in women with obesity and HFpEF [132]. Myocardial ischemia due to CMD may increase cardiac afterload and thereby contribute to the pathogenesis of HFpEF [132,133]. Although HFpEF and microvascular/endothelial dysfunction are unequivocally associated, a causal relationship has not been established [134]. 

### 4.2. Obesity

#### 4.2.1. Coronary Artery Disease

Obesity contributes to coronary atherosclerosis through multiple mechanisms including lipoprotein metabolism with the formation of intermediate-density lipoproteins (IDL) that undergo hepatic conversion to low-density lipoproteins (LDL) particles that accumulate with chylomicrons remnants (CMs) and oxidize in the subendothelial space of large arteries [135]. Both LDL and CMs are taken by macrophages and develop foam cells in atherosclerotic plaques. The uptake, entry, and retention of LDL in the vessel wall are key steps in the development of atherosclerosis. Obesity contributes to atherosclerosis through heightened systemic inflammation in addition to dyslipidemia, elevated triglyceride-rich lipoprotein cholesterol, low high-density lipoprotein, HT, and insulin resistance [136]. In addition to innate immunity, adaptative immunity aggravates atherogenesis through T-lymphocytes. With abundant inflammatory and proinflammatory markers, increasing visceral adiposity promotes the risks of atherosclerotic events [136]. The demonstration that obesity increases by twofold the inflammatory response in mice with atopic dermatitis supports the paradigm that visceral adiposity-mediated low-grade systemic inflammation hastens the development of atherosclerosis in humans [137,138]. In addition, obesity-associated low-grade inflammation mediates the development and progression of arterial stiffening through impaired collagen synthesis and increased degradation [103,139]. Perivascular adipose tissue (PVAT) that adjoins EAT is atherogenic and may contribute to atherosclerosis in patients with obesity [140]. Obesity hastens coronary atherosclerosis in adolescents and abdominal adiposity increases the risk of acute coronary events in young men [141,142]. 

#### 4.2.2. Microvasculature

Obesity leads to coronary microvasculature dysfunction (CMD) that contributes to HFpEF as well as global microvascular dysfunction that contributes to PHT, chronic kidney disease (CKD), and dementia [143]. Further, reduced coronary microvascular density decreases maximal myocardial blood flow in patients with BMI ≥ 30 [144]. Coronary microvascular dysfunction assessed by CFR is independently associated with poor outcomes in obesity [145]. Organ perfusion is altered via vasomotor changes, inflammation, and insulin resistance. Adipocyte enlargement reduces the O_2_ diffusion distance and promotes hypoxia which in turn decreases adiponectin release and increases leptin release from VAT [143]. Microvasculature dysfunction also affects the AT. Adipose-tissue blood flow increases after a meal in lean but not in overweight subjects [143]. Conversely, caloric restriction reduces endothelial NO synthase (ENOS) expression and activity in AT [143]. 

#### 4.2.3. Coronary Vascular Endothelium

**Figure 2:**
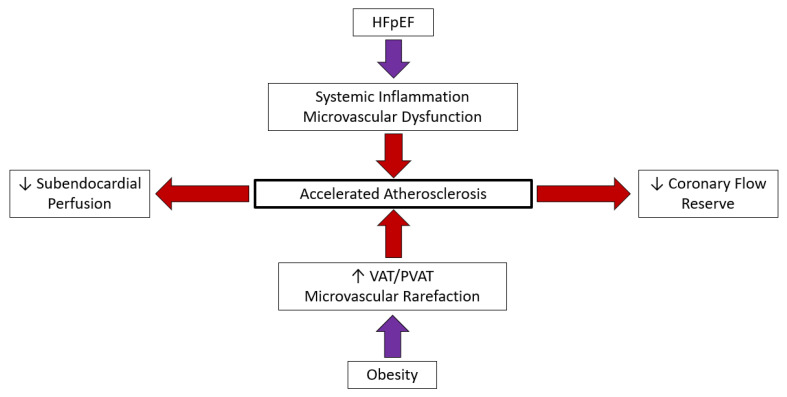
Circulation and HFpEF: HFpEF—Heart failure with preserved ejection fraction, VAT—Visceral adipose tissue, PVAT—Perivascular adipose tissue.

Obesity is an independent predictor of coronary endothelium dysfunction [146,147]. Impairment of endothelium-related coronary vasomotion progresses to impairment of the total coronary vasodilator capacity in patients with obesity [148]. The vascular endothelium plays a role in the regulation of metabolic homeostasis as endothelium dysregulation directly contributes to the development of metabolic disorders [149]. Dysfunctional AT-endothelial cell (EC) interaction may induce endothelium dysfunction leading to CAD in obesity [150]. Microvascular rarefaction appears to be organ specific. Whether organ specificity is related to the inherent characteristics of endothelial cells or the local parenchymal environment remains to be determined [151]. Obesity seems to affect endothelial cells in an organ and subtype-specific manner rather than in a global manner [152] (Figure 2).

## 5. Renal Function

### 5.1. Preserved Ejection Fraction Heart Failure

With continuous retention of water and salt as a cardinal component, HFpEF may be the quintessential renal problem [153]. Renal impairment contributes to the downward course of HFpEF and CKD, and when associated with systemic inflammation and increased oxidative stress, may enable incipient HFpEF [154,155]. The relationship between CKD and HFpEF is bidirectional as HFpEF is the preponderant cardiovascular condition in patients with advanced CKD and end-stage kidney disease (ERSD) [156,157]. In the Prospective Comparison of ARNI with ARB on the Management of HFpEF (PARAMOUNT) study, CKD (defined as estimated glomerular filtration rate [eGFR] ≥30 and ≤60 mL/min/1.73 m^2^, high urinary albumin-to-creatinine ratio [UACR] or both) was highly prevalent and associated with LV remodeling [158]. Albuminuria in patients with well-characterized HFpEF correlates with RV remodeling and worse outcomes [159]. Further, African Americans with CKD and PHT were at increased risk of fatal outcomes, and HFpEF hospitalization in the Jackson Heart study [160]. In addition to increased inflammation and oxidative stress, CKD may mediate the development of HFpEF through erythropoietin deficiency that lowers nitric oxide (NO) availability and impairs endothelial microvascular function [161]. Low NO decreases renal blood flow and eGFR, further exacerbating renal dysfunction [162]. 

### 5.2. Obesity

Obesity leads to glomerulopathy independent of HT and T2D through low-grade systemic inflammation, insulin resistance, intrarenal activation of the renin–angiotensin–aldosterone system (RAAS), endothelial dysfunction, and lipotoxicity [163]. Obesity-related glomerulopathy (ORG) is a distinct renal entity that entails proteinuria, progressive glomerulosclerosis, and functional decline [164]. Three pathways mediate obesity-induced renal injury: hemodynamic, AT-related, and insulin resistance/hyperinsulinemia [165]. Obesity-related hyperfiltration causes renal tubular damage by reducing the salt load in the macula densa and activation of the tubule-glomerular feedback [163,166]. Increased renal sinus and perirenal fat enhances NaCl tubular reabsorption and, through the macula densa feedback, renal blood flow and GFR [167]. Hypertrophied AT secretes and releases proinflammatory cytokines, including leptin, which drives renal dysfunction [168]. Leptin activates the sympathetic nervous system (SNS) and increases oxidative stress through fatty-acid oxidation. Adipokines and cytokines released by hypertrophied adipocytes and hypoxia-related insulin insensitivity promote insulin resistance and, in turn, hyperinsulinemia that affects podocyte function and glomerular barrier selectivity, leading to proteinuria [165].

The incidence of CKD rises when patients with BMI ≥ 30 develop metabolic abnormalities and progress from metabolically healthy to unhealthy obesity [169,170,171,172]. Obesity is an independent risk factor for ESRD with a relative risk of 3.57 when BMI is ≥30 and 7.07 when BMI is ≥40 compared to when BMI is ≤25 [173]. Over a follow-up of 30 years, midlife obesity increased the risk of incident renal failure and replacement therapy in all sex and race subgroups except for White men in the ARIC Study [174].

**Figure 3:**
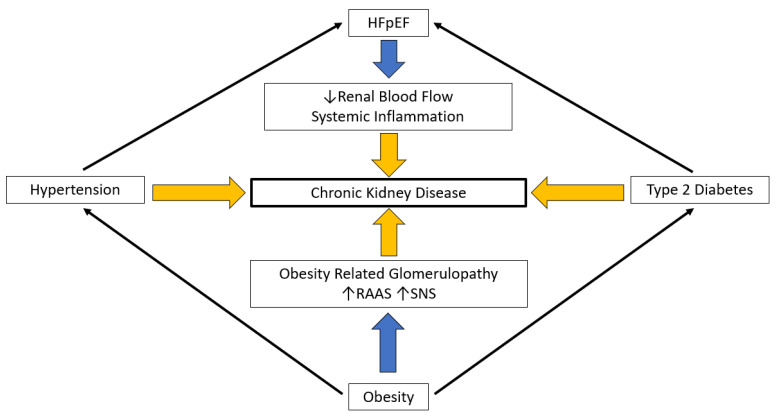
Renal function and HFpEF: HFpEF—Heart failure with preserved ejection fraction, RAAS—Renin angiotension aldosterone system, SNS—Sympathetic nervous system.

In brief, obesity-related CKD undoubtedly accelerates the symptomatic decline in patients with HFpEF. Renal function correlates inversely with BMI and the duration of obesity and positively with LVDD [175]. When evolving rapidly, obesity-related kidney disease may underlie the premature development of HFpEF syndrome (Figure 3).

## 6. Moving Forward: Metabolic Bariatric Surgery in Patients with Obesity and Preserved Ejection Heart Failure

The current pharmacologic treatment of HFpEF centers on the control of cardiac pre- and afterload with loop diuretics, sodium-glucose cotransporter type 2 inhibitors (SGLT2i), and arterial vasodilators like angiotensin-converting enzyme inhibitors (ACEIs), angiotensin receptor blockade (ARBs), or calcium channel antagonists [176]. Except for tight control of HT with a systolic BP goal of <130 mmHg, current pharmacologic treatment does not target the reversal of LV remodeling. Whether obesity amplifies HT-mediated LV remodeling or initiates LV remodeling, long-term therapeutic effectiveness likely depends on the reversal of LV remodeling in patients with severe obesity and HFpEF. Although challenged by novel antiobesity medications, MBS remains the most effective intervention for ≥20% sustained loss of body weight [177,178]. One expects MBS to significantly decrease LVM and alleviate LVDD in patients with severe obesity and HFpEF (Figure 1).

### 6.1. Metabolic Bariatric Surgery and Left Ventricular Mass

Cardiac MRI is the gold-standard imaging modality for quantifying LVM (indexed to height in obesity) and is, therefore, the optimal modality for assessing the cardiac effect of MBS in subjects with obesity [179,180]. Only a few studies examined the cardiac effects of MBS with CMR in patients with severe obesity. Eight adult studies comprising 170 subjects with severe obesity appraised LVM by CMR before and after MBS (Table 2) [181,182,183,184,185,186,187,188]. The mean reduction in LVM was 11.1% in these studies. Improvement in the peak LV filling rate correlated with LVM reduction in one study, and LVDD improved in one of the three patients with known LVDD in the other study. Past echocardiographic studies on MBS patients report that reduction in LVM does not consistently translate into LVDD improvement [185,189,190]. An echocardiographic-driven study on MBS and LVDD demonstrated an improvement in LVM and tissue-doppler-derived mitral annular early-diastolic velocity (e’), without a statistically significant difference in echo-derived left atrial filling pressure (defined as E/e’) [191].

### 6.2. Incidence of Heart Failure after Metabolic Bariatric Surgery

The modest effect of MBS on LVM contrasts with the markedly lower incidence of HF in the years that follow MBS. The hazard ratios for incident HF admission after MBS were 0.54 (95% confidence interval (CI) 0.36–0.82) and 0.37 (95% CI 0.29–0.46) in the Sweden National Patient Registry and Scandinavian Obesity Surgery Registry, respectively [192,193]. A hazard ratio of 0.44 (95% CI 0.31–0.62) after MBS for major adverse cardiac events in patients with HF from Ontario, Canada corroborates the Swedish and Scandinavian findings [4]. Better control of BP and T2D, lower systemic inflammation, intermuscular fat and neurohormonal activation, greater adipokines levels (Leptin, adiponectin) vascular endothelium function and physical activity as well as fat redistribution and slower deterioration of renal function may underlie the beneficial effects of MBS on HF hospitalization rather than via reduction in LVM or improvement in LVDD [19,194,195].

## 7. Selection of Patients for Metabolic Bariatric Surgery

### 7.1. Duration of Obesity and Preserved Ejection Fraction Heart Failure

Obesity is twice as common in <55 years old and <65 years old Asian patients with HFpEF as in their ≥65 years old counterparts [196]. Obesity is also more prevalent in patients < 65 years than in patients ≥ 65 years old patients hospitalized for HFpEF [197]. Patients < 55 years old had a greater BMI and more concentric LV hypertrophy than patients ≥ 85 years old in three large randomized HFpEF trials [198]. The prevalence of obesity in HFpEF patients varies among ethnicities. Young HFpEF patients in the New York Heart Failure Registry were predominantly Black non-Hispanic, and their BMIs were greater than that of patients with other racial backgrounds [199]. African Americans with HFpEF had a mean BMI of 37 in the Urban Baltimore Community [200,201].

The duration of obesity may be as or more detrimental to patients than the degree of obesity [202]. Three decades ago, a cross-sectional study of 30 patients uncovered a positive relationship between the duration of obesity and LV wall thickness [203]. With time EAT accumulates and becomes inflamed, contributing to LV hypertrophy and impairing LV diastolic function [40]. Younger patients experience a greater reduction in EAT with pharmacologic obesity therapy with glucagon-like peptide 1 receptor antagonists (GLP-1 RAs) and SGLT2is [204]. Duration of obesity correlates positively with LVM and LVDD in patients with severe obesity [205]. Further, the duration of morbid obesity is the strongest predictor of incident HFpEF in normotensive patients without overt cardiovascular disease [206]. Left ventricular diastolic dysfunction is congruent with long-lasting severe obesity and is associated with reduced eGFR [175]. Independently of a healthy or unhealthy status, the duration of severe obesity was a major risk of incident HF in the Nord-Trondelag Health (HUNT) study [207]. The duration of total and abdominal obesity was a stronger predictor of cardiac remodeling than the severity of obesity in the Coronary Artery Risk Development in Young Adults (CARDIA) study [61]. Nonetheless, a rapid increase in adiposity metrics, such as BMI and waist circumference (WC), increased the risk of hospitalization for HF in the biracial ARIC study [208].

A steadfast link ties longstanding obesity to cardiovascular risk, outcome, and all-cause mortality [209]. When assessed by excess BMI and WC years, longstanding obesity predicted cardiovascular disease risk in the Coronary Artery Risk Development in Young Adults (CARDIA) study [210]. Greater exposure to excess BMI and WC years portended higher rates of T2D, and levels of BP, insulin, triglycerides, and total cholesterol [210]. Each incremental 100-excess BMI years led to subclinical myocardial damage and thereby increased HF risk in the predominately biracial ARIC study [211].

### 7.2. Obesity Exposure, Cardiac Remodeling, and Cardiovascular Outcomes

Cardiovascular MRI allowed for a better understanding of the time course of obesity-mediated cardiac remodeling in HF [71]. T1 mapping showed promise in the detection of myocardial structural abnormalities [191]. Adipose tissue shortens myocardial T1 relaxation time and severe obesity is associated with low native myocardial T1. On the other hand, a high native T1 is associated with myocardial fibrosis and linked to an increased risk of HF. In brief, low T1 native reflects subclinical myocardial lipid accumulation and early-stage obesity-mediated myocardial remodeling whereas high native myocardial T1 reflects fibrotic degeneration and late-stage obesity-mediated LV remodeling [71]. Large loss of body weight may not reverse obesity-mediated cardiac remodeling in patients with high native myocardial T1. In other words, patients with chronic fibrosis may not experience a reversal of cardiac remodeling despite large weight loss. The time course of eGFR decline parallels that of cardiac remodeling in patients with high BMI. The odds of incident CKD increased by 21% for each unit increment in BMI after adjustment for T2D, smoking, and baseline eGFR over 18 years in the Framingham Offspring Study [212]. Last, obesity-mediated LVDD and the duration of obesity hastens the decline of renal function [175].

**Figure 4:**
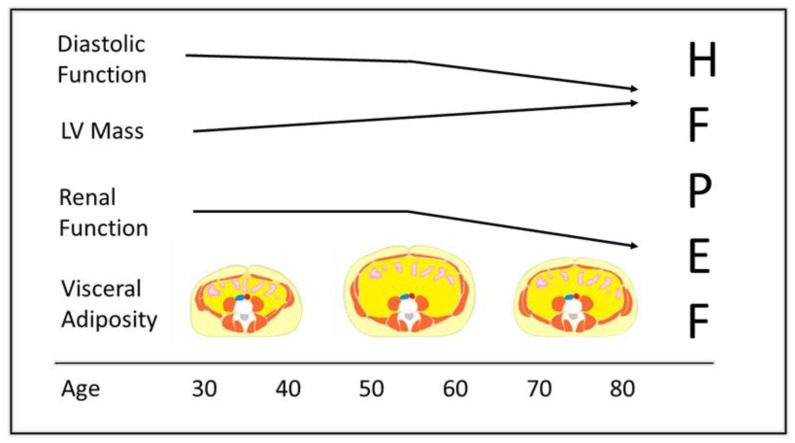
Left ventricular diastolic function, left ventricular mass, and renal function trajectories in relation to visceral adiposity in early, middle, and late adulthood. Accumulation of visceral adiposity in middle adulthood heightens an age-related increase in left ventricular mass and a decline in renal and diastolic function. The late-adulthood reduction in visceral adiposity may be spurious due to the attrition of subjects with great visceral adiposity [215,216].

In brief, young (<55 years) patients with obesity and HFpEF are prime candidates for evaluation of the optimal effects of MBS on LV remodeling. The higher BMI and greater concentric LV hypertrophy in young (<55 years) patients with HFpEF compared to their older (>65 years) counterparts hint at a greater pathogenic role of obesity in younger patients [196]. Further, with less cardiac fibrosis, younger patients are more likely to experience a reversal of LV remodeling than elderly patients [213]. Although they were 8–10 years younger than their normal or overweight counterparts, the age of patients in current investigations of the obese phenotype of HFpEF still averaged 65 years [29,214]. The selection of patients >65 years old may thwart the potential therapeutic benefit of MBS on cardiac remodeling (Figure 4).

## 8. Conclusions

Concentric left ventricular remodeling or hypertrophy commonly underlies the development and progression of preserved ejection heart failure syndrome. Current treatment of left ventricular remodeling in patients with preserved ejection heart failure consists of tight control of systemic blood pressure. The coexistence of severe obesity and preserved ejection heart failure provides a unique opportunity to evaluate the effects of a large loss of excess body weight via metabolic bariatric surgery on left ventricular remodeling and functional capacity in patients with the obese phenotype of preserved ejection fraction heart failure.

## Figures and Tables

**Table 1:** Six-minute walk test distance according to body mass index (ref ---to---).

BMI (kg/m^2^)	30–35	35–40	40–45	45–50
Distance (meters)	561	475	455	417

Adapted from Donini et al. 2013 [87]. 6-min walk test distance per BMI.

**Table 2:** Left Ventricular Mass Reduction Following Metabolic Bariatric Surgery.

Study	Study Population	Percent Weight Loss	LVM Decrease	Length of Study
Jhaveri et al. [183]	17	32%	32%	17 months
De Witte et al. [184]	13	31.2%	15.2%	12 months
Leichman et al. [185]	22	15%	7.8%	3 months
Rider et al. [182]	13	19%	10%	12 months
Henry et al. [181]	62	29%	12%	1030 Days (33.8 months)
Gaborit et al. [186]	23	14.4%	14.5%	6 months
Schneiter et al. [187]	11	54.7% *	10%	15.4 months
Van Schinkel et al. [188]	9	17.2%	4.9%	16 weeks (3.7 months)
Total/Mean	170 ^α^	22.5% ^Ϯ^	13.3% ^β^	12.9 months ^β^

* Reported as percent excess weight loss, ^α^ Total, ^Ϯ^ Excluding Schneiter et al. [187], ^β^ Mean.

## Data Availability

Not applicable.
